# Morphological variation on isolated populations of *Praocis* ( *Praocis* ) *spinolai*

**DOI:** 10.1093/jis/14.1.11

**Published:** 2014-01-01

**Authors:** Hugo A. Benítez, Jaime Pizarro-Araya, Raffaella Bravi, María-José Sanzana, Fermín M. Alfaro

**Affiliations:** 1 Faculty of Life Sciences, University of Manchester, Michael Smith Building, Oxford Road, Manchester M13 9PT, UK; 2 Instituto de Alta Investigación, Universidad de Tarapacá, Casilla 7-D Arica, Chile; 3 Laboratorio de Entomología Ecológica, Departamento de Biología, Universidad de La Serena, Casilla 599, La Serena, Chile; 4 Environmental Biology Department, University Roma Tre, V. le G. Marconi 446, 00146, Rome, Italy; 5 Departamento de Zoología, Facultad de Ciencias Naturales y Oceanográficas, Universidad de Concepción, Concepción, Casilla: 160-C, Chile; 6 Laboratorio de Genética y Evolución, Departamento de Ciencias Ecológicas, Facultad de Ciencias, Universidad de Chile, Santiago, Chile

**Keywords:** coastal desert, epigean tenenebrionids, geometric morphometrics, Pingüino de Humboldt National Reserve

## Abstract

In this study, the morphological variations of four geographically isolated populations of *Praocis* ( *Praocis* ) *spinolai* Gay & Solier (Coleoptera: Tenebrionidae) in the transitional coastal desert, Chile, were studied. The study was conducted in the coastal area of Punta de Choros and Los Choros-Archipelago, which includes three islands: Choros, Damas, and Gaviota. 113 specimens of the species *P.* ( *P.* ) *spinolai* belonging to the four locations sampled were collected analyzed with geometric morphometrics techniques to explore the pattern of shape variation on the different isolated environments. The principal component analysis revealed a well-defined pattern of variation between the populations analyzed. Moreover, differences between populations emerged also from the canonical variation analysis and were confirmed by the Procrustes ANOVA. All analyses performed confirmed the existence of a pattern of variation, due to the isolation of the populations and to environmental effects. The islands are subject to more arid pressures than the continent, where there is a more stable environment and the presence of coastal wetlands and the coastal range of mountains act together and enable fog condensation. This study indicates the existence of a clear pattern of variation, which indicates an evolutionary trend among the population examined.

## Introduction

Over the last century, research on islands has continued to advance the understanding of the evolutionary process ( Emerson 2008 ). Island archipelagos provide unique scenarios for studying the roles of geography and ecology in driving population divergence and speciation while playing a crucial role in the diversification of biotas. Oceanic islands also have long been recognized as natural laboratories for the study of evolutionary processes ( Mayr 1967 ; Grant 1998 ; Losos and Ricklefs 2009 ; Mila et al. 2010 ).

The Pingüino de Humboldt National Reserve is located on the coastal border between Huasco (Atacama Region) and Elqui (Coquimbo Region) provinces in Chile, and it comprises a total area of 859.3 ha. It was created in 1990 and is a part of the country’s National System of Protected Wild Areas. A portion of the reserve encompasses the Choros Archipelago, which includes the islands of Choros, Damas, and Gaviota. These islands are located on the northwestern end of the Punta Choros area, Coquimbo Region ( Castro and Brignardello 2005 ), and constitute a peculiar insular ecosystem. The three islands are located in an area within the transitional coastal desert (25–32º Lat S), the latter of which is characterized by the presence of an unusually-species-rich arthropod fauna ( Cepeda-Pizarro et al. 2005 ; Valdivia et al. 2008 , 2011; Pizarro-Araya et al. 2008 ; Alfaro et al. 2009 ; Alcayaga et al. 2013 ), endemism ( Jerez 2000 ; Pizarro-Araya and Flores 2004 ; Ojanguren-Affilastro et al. 2007 ; Pizarro-Araya et al. 2012a ,b; Ojan-guren-Affilastro and Pizarro-Araya in press ; Laborda et al. 2013 ), and restricted distribution ( Pizarro-Araya and Jerez 2004 ; Agusto et al. 2006 ; Alfaro et al. 2013 ).

It is well known that adaption over time to a specific environment is the result of both environmental pressures and geographic distances, affecting geographic micro-environments at a local scale and thus their associated flora and fauna ( Alibert et al. 2001 ; Cepeda-Pizarro et al. 2003 ; Benítez et al. 2008 ; Benítez 2013 ). Moreover, it is well documented that adverse temperatures, nutritional stress, presence of chemicals, population density, and many other factors that cause stress during development can lead to an increase in the presence of morphological asymmetries as a result of high intraspecific variation (e.g., Rettig et al. 1997 ; Benítez et al. 2008 ; Benítez 2013 ). Therefore, it is expected that when environmental conditions change, organisms and populations should adapt to the new conditions ( Clarke 1993 ). In this context, adaptive variation plays a major role because it reflects historical evolution and determines the population’s phenotypic response. Floate and Fox (2000) and Piscart et al. (2005) suggested that the degrees of phenotypic disturbance reflect the ability of an individual to overcome the effects of stress. In fact, in epigean arthropods the more symmetrical individuals would have a greater survival chance than those with any level of asymmetries.

Among epigean arthropods, Tenebrionidae (Coleoptera) constitute a characteristic group of the arid and semiarid ecosystems fauna ( Cloudsley-Thompson 2001 ; Deslippe et al. 2001 ). Knowledge of Tenebrionidae in the transitional coastal desert is limited to the reports by Cepeda-Pizarro et al. (2005) , who documented the presence of 20 species belonging to 14 genera for the northern area between 27 and 30º S. Alfaro et al. (2009) documented the presence of 14 species in Pingüino de Humboldt National Reserve, arranged in eight genera and six tribes, from which seven species were common to the archipelago and five genera were reported for the first time as occurring in insular habitat islands: *Psectrascelis* Solier, *Entomochilus* Solier, *Diastoleus* Solier, *Sco-tobius* Germar, and *Thinobatis* Eschscholtz. *Gyriosomus granulipennis* Pizarro-Araya & Flores was recorded as endemic to the Choros Island ( Pizarro-Araya and Flores 2004 ; Alfaro et al. 2009 ; Pizarro-Araya et al. 2012a ) and *Praocis (Praocis) spinolai* Gay & Solier was the most abundant species among the beetles registered at the three islands ( Alfaro et al. 2009 ). Because these islands represent one peculiar insular ecological unit within the transitional coastal desert, the aim of this study was to evaluate the island effect of isolated geographic areas on the morphological differentiation between four populations of *Praocis (Praocis) spinolai* using a geometric morphometrics approach.

## Materials and Methods

### Study area

**Figure 1.**
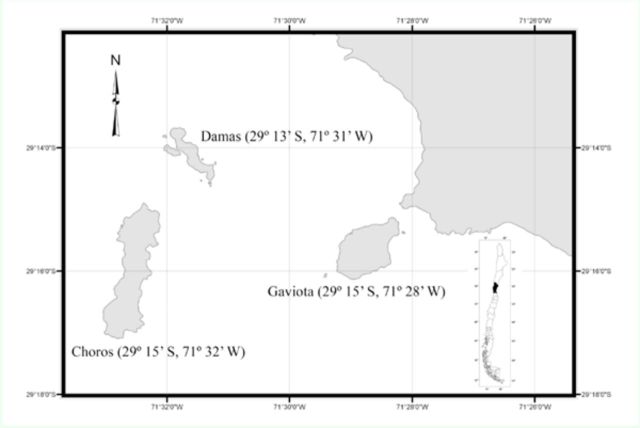
Map of the mainland of Los Choros indicating the study area and the sampling sites, the islands of Choros, Damas, and Gaviota (Coquimbo Region, Chile). High quality figures are available online.

The study was conducted in the coastal area of Punta de Choros (29º 15’ S, 71º 26’ W) and Los Choros Archipelago (29º 32’ S, 67º 61’ W), which includes three islands: Choros (29º 15’ S, 71º 32’ W), with a surface of 322 ha, Damas (29º 13’ S, 71º 31’ W), with a surface of 56 ha, and Gaviota (29º 15’ S, 71º 28’ W), with a surface of 182 ha. This coastal desert area is located ~114 km north of La Serena, Coquimbo Region, Chile ( Figure 1 ). The area has a Mediterranean type climate with morning fog ( Di Castri and Hajek 1976 ).

### Sampling

The data on taxonomical composition were collected by means of pitfall traps set up in the continental and insular ecotopes. The traps were placed in four contrasting pedo-logical units for the continental area, and three for each island. The continental zone was represented by the coastal area of Punta de Choros, which is a coastal desert zone. Four environments were selected for this area, namely coastal steppe, coastal dune, coastal wetland, and interior coastal steppe. These environments were characterized by sandy soil scarcely developed and flat scrubland. The island area was represented by three sites for each one of the islands in the archipelago Los Choros. The sites selected for the island system were characterized by cliffs with stony soils (Isla Choros) and sandy soils (Damas and Gaviota) with poor vegetation ( Castro and Brignardello 2005 ). Two plots (4 **x** 5 m each) were established in each ecotope, and 20 pitfall traps were arranged at 1-m intervals in each plot. Each trap consisted of a plastic jar (70.4 mm diameter, 102 mm height) filled to two-thirds capacity with a 3:7 mixture of formalin (10%) and water with detergent. The traps were active for three days during four months (June, August, October, and November) in 2005 and three months (August, October, and December) in 2006. The material collected was retrieved, cleaned, and preserved in alcohol (70%) until processing. Sampled specimens are now stored in the collection of the Laboratorio de Entomología Ecológica at the Universidad de La Serena, La Serena, Chile (LEULS).

### Morphometric analysis

**Figure 2.**
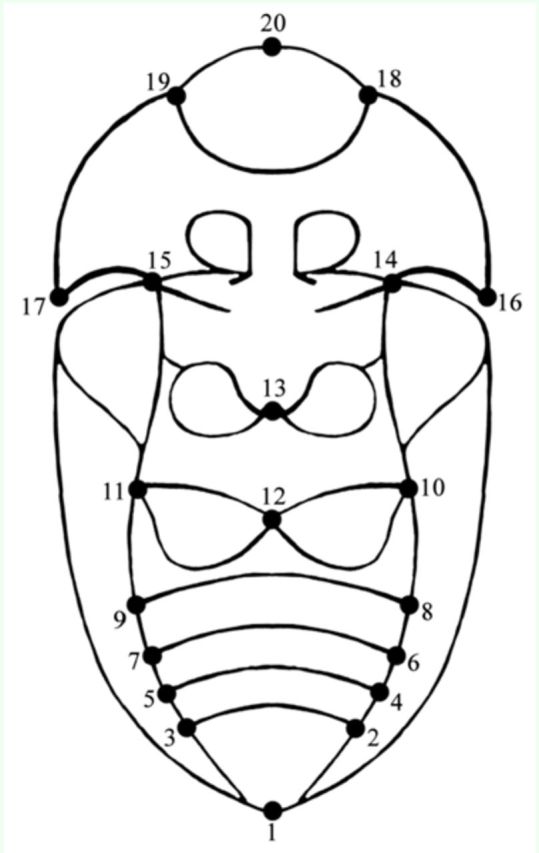
Indication of 20 landmarks in the ventral view of *Praocis (Praocis) spinolai.* 1: pygidium, 2: right lateral vertex of abdominal segment 4, 3: left lateral vertex of abdominal segment 4, 4: right lateral vertex of abdominal segment 3, 5: left lateral vertex of abdominal segment 3, 6: right lateral vertex of abdominal segment 2, 7: left lateral vertex of abdominal segment 2, 8: right lateral vertex of abdominal segment 1, 9: left lateral vertex of abdominal segment 1, 10: right lateral vertex of metastern, 1 1: left lateral vertex of metastern, 12: mean point of metastern, 1 3: lower mean point of mesostern, 14: right vertex of pronotal epimere, 15: left vertex of pronotal epimere, 16: right pronotal posterior angle, 17: left pronotal posterior angle, 18: right vertex of lip, 19: left vertex of lip, 20: mean point of head between mandibles. High quality figures are available online.

A total of 117 selected specimens of *P. (P) spinolai* were used for the morphometric analyses. Fifty-five individuals were analyzed from the continental ecotope and 58 from the island ecotope (33 Gaviota, 25 Choros, and 4 Damas). The ventral side of each individual was photographed using a Nikon Coolpix L120 digital camera (14 megapixel, www.nikon.com ). Twenty landmarks were digitized (anatomical homologous points) on every picture with TpsDig 2.10 ( Rohlf 2006) ( Figure 2 ). All analyses were then run using MorphoJ software version 1.05a ( Klingenberg 2011 ).

Once the Cartesian x-y coordinates were obtained for all landmarks, the shape information was extracted with a full Procrustes fit (generalized Procrustes analysis, Rohlf and Slice 1990 ; Dryden and Mardia 1998 ), taking into account the object symmetry of the structure. Procrustes superimposition removes the information of size, position, and orientation, and standardizes each specimen to unit centroid size (obtained as the square root of the summed squared Euclidean distances from each landmark to the specimen centroid) and provides an estimation of the size of the studied structure ( Dryden and Mardia 1998 ). For studies of object symmetry, reflection is removed by including the original and mirror image of all configurations in the analysis and superimposing all of them simultaneously ( Klingenberg et al. 2002 ).

Shape variation was analyzed in the entire dataset with principal component analysis (PCA) based on the covariance matrix of symmetric and asymmetric components of shape variation. The first one is the average of left and right sides and represents the shape variation component, whereas the asymmetric component represents the individual left-right differences ( Klingenberg et al. 2002 ).

Differences between locations were assessed using canonical variate analysis, a multivariate statistical method used to find the shape characters that best distinguish among multiple groups of specimens. Because of the lack of specimens for the Damas population, the analysis was run only for the other three populations. The results were reported as Mahalanobis distance and Procrustes distances and the respective *p* -values, after a permutation test that runs 10,000 permutations.

Finally, Procrustes ANOVA for size and shape and MANOVA analyses assessed for studies on object symmetry were performed to evaluate if the observed differences in the sample were due to real differences in the populations examined.

## Results

**Figure 3.**
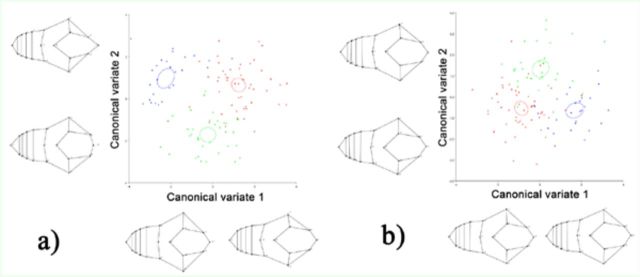
Canonical variate analysis of three of the four isolated populations of *Praocis (Praocis) spinolai:* Gaviota (green), Choros (blue), and mainland (red). In the figure are shown the first two canonical variate compenents’ axes with shape deformation images associated. (A) Canonical variate analysis for the symmetric component, (B) canonical variate analysis for the asymmetric component. High quality figures are available online.

The PCA for the symmetric component (individual variation) showed differences between the three populations analyzed. The first two PCs accounted for 55.37% (PC1 + PC2 = 33.33% + 22.04%) of the total shape variation and provided a reasonable approximation of the total amount of variation. The other PC components accounted for no more than 12% of the variation each. The PCA analyses for the asymmetry component (left-right asymmetries) showed differences between populations as well. The first two PCs accounted for 52.33% (PC1 + PC2 = 38.84% + 13.49%) of the total shape variation, and the other PCs accounted for no more than 9% of the variation each. According to PCA, canonical variate analysis showed significant differences in both symmetric and asymmetric components between the three popula-populations examined and after permutation test (10,000 permutation runs) ( Table 1 , Figure 3 ). Finally, Procrustes ANOVA for size did not show significant differences between populations *(F =* 1.37, *p* < 0.2545). Procrustes ANOVA for shape showed differences between populations *(F =* 3.05, *p* < 0.0001) and high differences among individuals emerged ( *F* = 5.79, *p* < 0.0001). MANOVA tests, for both symmetric and asymmetric components, confirmed these results (Pillai’s trace = 1.09, *p* < 0.0001; Pillai’s trace = 0.78, *p* < 0.0001, respectively).

**Table 1.**
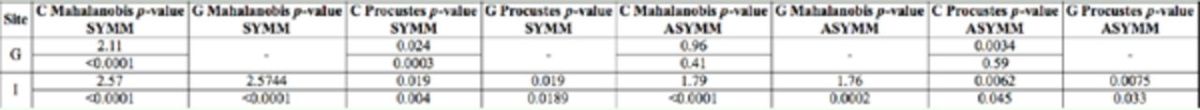
Results of the canonical variate analysis with Mahalanobis and Procrustes distances and the respective *p* -values for the symmetric and asymmetric components of the variation.

G: Gaviota, I: Choros, C: Mainland

## Discussion

Morphological differences in both individual and populations were found in this study. The populations examined are well-separated, indicating the existence of an evolutionary pattern.

The observed differences could be due to the isolation of the populations and climatic-environmental effects, as the islands are subjected to more arid conditions than the continental area, as the continent has a more stable environment due to the presence of coastal wetlands and the coastal range of mountains, enabling fog condensation ( Cepeda-Pizarro et al. 2005 ; Valdivia et al. 2011 ). Under stochastic processes and environmental stress, the isolated and small populations suffer more than the large interconnected populations, as studies have shown the populations more affected by losses of genetic variability are small and isolated populations ( Frankham et al. 2001 ; Allendorf and Luikart 2007 ). These losses are often accompanied by a negative impact on individual fitness ( Reed and Frankham 2003 ). In general, the reductions in viability are reflected in morphologic traits or asymmetry. The results presented here indicate that morphological variations and the variation among sampling sites were mainly due to differences in shape. It is frequently suggested that morphological variation of individuals may be strongly dependent upon unfavorable environmental conditions ( Adams and Funk 1997 ; Tatsuta et al. 2001 ). In fact, individuals under environmental noise could develop any kind of asymmetries ( Van Valen 1962 ).

Although the differences in body shape observed were not obvious, individuals from the mainland had thinner bodies than those from the islands. It has been reported that a climate with high relative humidity and constant temperatures promotes a thinner subelytral cavity, thus this result was expected for the mainland ( Draney 1993 ; Duncan 2002 , 2003).

The individuals of the different islands had more pronounced morphological variation, which may be a consequence of the heterogeneity of the environment in this area (higher variation in temperature ranges, which leads to thicker subelytral cavities). Regarding habitat heterogeneity, Fattorini (2009) analyzed the diversity of Tenebrionidae in contrasting ecotopes in the Mediter-Mediterranean island of Santorini (Greece) and concluded that differences in the composition of tenebrionid assembly could be attributed to climate and substrate type, indicating that these are the most important factors regulating the species diversity.

Observations in other latitudes (Lute desert, Central Iran) have found that morphological variations (e.g., pronotum size) in disjunctive populations of psammophilic Tenebrionidae could be related to factors such as temperature and food availability ( Taravati et al. 2009 ). Zachariassen et al. (1987) showed that Tenebrionidae exhibited the lowest water loss rate compared to other desert insects. The authors proposed that these Tenebrionidae use three major physiological characteristics to conserve water: reduction of cuticular water permeability, reduction of breathing water loss due to subelytral cavity, and reduction of metabolic rate.

Due to the isolation that affects the populations examined, the gene flow has been interrupted between them, and the group shows a particularly high plasticity in the capacity to withstand differences and environmental pressures imposed in each particular environment (Palmer 2000). This capacity was reflected in the high morphological plasticity that emerged and indicates that the populations are evolving.
